# Obstacles to using online health services among adults age 50 and up and the role of family support in overcoming them

**DOI:** 10.1186/s13584-020-00398-x

**Published:** 2020-08-21

**Authors:** Y. Mizrachi, S. Shahrabani, M. Nachmani, A. Hornik

**Affiliations:** 1 Sociology and Anthropology Department, The Max Stern Yezreel Valley College, 1930600 Emek Yezreel, Israel; 2 Economics and Management Department, The Max Stern Yezreel Valley College, 1930600 Emek Yezreel, Israel; 3 Department of Sociology and Anthropology, The Max Stern Yezreel Valley College, 1930600 Emek Yezreel, Israel; 4 Department of Psychology, Bar Ilan University, 52900 Ramat Gan, Israel

**Keywords:** Online health services, Compliance, Older adults, Israel

## Abstract

**Background:**

Using Online Health Services (OHS) could benefit older adults greatly and could also reduce the burden on the health system. Yet invisible obstacles or barriers appear to impede mass adoption of these services among this population group. The aim of the current research is to provide a qualitative picture of these invisible obstacles and to profile their main features, with special attention to the role of family members in supporting OHS use among this population group.

**Methods:**

This qualitative study entailed a series of in-depth, semi-structured, open phone interviews conducted with 31 individuals age 50 and up in Israel, who constituted a sample of OHS users and non-users among older adults.

**Results:**

Four major themes and primary observations emerge from our data:

1. While older adults are aware of OHS to some extent, they often do not fully understand the specific benefits of using these services;
2. Older adults need to acquire much more experience with OHS use. OHS user interfaces still have a long way to go for older adults to feel comfortable using them. People age 50 and up seem to be less concerned about privacy and security issues than about seemingly more trivial issues such as recovering forgotten passwords;
3. Family members can play key roles in helping older adults adopt OHS by providing technical support as well as encouragement;
4. Older adults have worthwhile recommendations for innovations and policy improvements that would facilitate wider adoption of OHS.

**Conclusions:**

The results of the current study reveal important nuances regarding the importance of awareness, user interface and experience for OHS use among older adults, as well as the critical role of family members in OHS adoption. Based on these findings, we recommend the following: expanding advertising on media channels to emphasize the benefits of OHS use; improving HMO websites to make them more user-friendly for older people; developing HMO-run community OHS guidance programs geared to older people to reduce the gap between required skills and user competencies, thus enabling older people to benefit from OHS use.

## Background

The term Online Health Services (OHS) is used as an umbrella term to encompass all online health-related actions, such as searching for essential medical information online, making doctor’s appointments online, obtaining the results of medical examinations and even remote telemedicine services. Older adults are most likely to be the primary beneficiaries of Online Health Services (OHS). Moreover, widespread OHS use among this population group also has the potential to reduce the burden on the health system. Yet paradoxically, despite the clear and overwhelming advantages of new internet and cyber-enabled technologies for older adults, invisible obstacles seem to impede mass adoption of these services among this population group. For example, in the United States the rate of OHS use was much lower among individuals age 65+ as well as among individuals with lower socio-economic status [1] than among younger individuals [2].

The current research seeks to describe these invisible obstacles and to profile their main features, with special attention to the level of support family members can provide older adults with respect to OHS use.

In Israel, people age 65 and above make extensive use of health services (e.g., an average of 11.2 annual visits to general and family physicians compared to an average of only 3.2 annual visits among the general population age 20 and above [3]). Yet according to the 2018 Statistical Abstract of Israel, only 51% of Israelis over the age of 65 use computers, compared to 72% of all adults over the age of 20. Among the population of older adults who do use computers, the primary uses are information searches (91%), e-mail (72%) and social networks (72%) [4].

In a previous study, we examined the degree of responsiveness and willingness to use different OHS among Israelis age 45+ and characterized the attitudes and main factors influencing their responsiveness [5]. That study entailed a telephone survey of a sample of 703 individuals constituting a representative sample of the Israeli population. The research questionnaire integrated the principles of the Healthcare Information Adoption Model [6, 7] based on the Technology Acceptance Model [8, 9] and included socio-demographic attributes as well. The results indicated that approximately half of adults age 45+ use the internet.^1^ Moreover, 78% of the surveyed internet users claimed to use at least one OHS (79% in the Jewish sector and 66% in the non-Jewish sector), while 22% of the internet users reported not using OHS at all. Most OHS use involved visiting Health Maintenance Organizations (HMO) websites to obtain administrative information, with only 17% using HMO websites for medical consultations. Among the main reasons respondents cited for not using OHS were that they found the services difficult to use, they had no need for OHS and they were unaware of these services. The study also found significant differences between populations according to socioeconomic attributes (e.g., gender and income). For example, higher rates of using forums to obtain medical information were found among women, native-born Israelis and individuals with average or above-average incomes. The results of our study [5] also indicated that 32% of older OHS users received help from their families. Furthermore, the percentage of those who receive help from their families was significantly higher in the non-Jewish sector (45%) than in the Jewish sector (30%). The results also showed that people’s intentions to use remote services were greater when family members encouraged online use.

The results of the above survey also indicated that frequency of OHS use rises as the following factors increase: perceived ease of use; extent of encouragement for OHS use; perceived reliability of online health services; and extent of exposure to advertising. The study also found that OHS use is much more prevalent among wealthy populations and that attitudes and extent of advertising exposure influence both OHS use and intended use. Several recommendations emerged from the study: 1) To boost OHS use, online health websites should be made more user-friendly to individuals over the age of 45 and to those from different language backgrounds and cultures. 2) Programs should be developed to teach HMO staff how to encourage patients to use OHS. 3) Media advertising should be expanded to encourage OHS use.

The large body of literature examining patterns of technology use, including OHS, among older adults distinguishes three generations of digital divide [10]. The first-generation digital divide refers to accessibility and user-friendliness and focuses on difficulties obtaining the information appearing on websites due to lack of computer access [11]. The second-generation digital divide centers on the “ability to use technology” [10]. This divide entails situations in which the technological means are available but users lack the requisite knowledge to use them effectively and derive maximum benefit from them. The third-generation digital divide derives from the second-generation digital divide and is referred to as the “divide of digital outcomes” [12]. This divide focuses upon inequalities in the benefits derived from ICT (information and communication technologies) usage.

A qualitative study conducted in the US [12] examined six groups of older adults who were early technology adopters. The study found that these adults want to make their own final health decisions, supported by input from health services providers. To this end, these older users want their health services providers to screen online information and select only information that is reliable. They use OHS frequently, but only for information-seeking and not for diagnosis, treatment or disease management. Another study from the US [13] showed that intergenerational interactions in which older adults were mentored by college students resulted in a significant drop in technophobia and major improvements in OHS literacy, self-efficacy and interest in technology.

Digital literacy is defined as possessing the set of skills necessary for using ICT [14]. Many older adults have a low level of digital literacy. They avoid technology because they are uninformed about it or unable to use it effectively. Since the internet is quickly becoming the main means of information dissemination, non-users or those with low levels of digital literacy are at a disadvantage.

Another type of digital literacy is eHealth literacy, defined as possessing the set of skills required for effectively seeking, finding, understanding and appraising information technology used for health and applying the knowledge gained to address or solve a health problem. These skills are part of basic eliteracy in the fields of health, science, media and computers [15]. The literature indicates that eHealth literacy is lower among older adults, those with lower SES and those with less computer experience [16]. eHealth literacy also has been found to be associated with attributes such as number of e-devices in the user’s possession, computer-related stress, and health knowledge and attitudes (including medical decision-making, health information sources and the like) [17]. In contrast, higher eHealth literacy has been found to be associated with more positive outcomes of internet searches in three domains: cognitive (e.g., health knowledge/information-gathering), instrumental (e.g., self-management of health needs and health behaviors) and interpersonal (e.g., communication with doctors) [12, 18, 19].

A study conducted in Israel showed that although the number of eHealth users has increased significantly over the years, a third of the older adult population does not use eHealth services despite having smartphones and internet access [18]. Other studies conducted in Israel showed that the use of smartphone eHealth applications helped older adults improve their health management and health status [19, 20]. Still other studies found that older adults who use customized online medical databases improved their knowledge about the medical topics they investigated [21].

Today online health services are undergoing continuous improvement to meet the constantly growing needs for OHS, especially among older adults. In addition, knowledge, capability and e-health orientation are continuing to increase among the newer generations of people age 50 and up. Therefore, updated research is needed to examine the barriers to OHS use among this population. Very few studies have examined OHS usage in Israel, and to the best of our knowledge most of these used quantitative methods. The current study adds to the existing literature by examining the current situation using the qualitative method of in-depth interviews to reveal the larger picture regarding OHS use among people age 50 and up in Israel.

While previous research provided a clear picture of the general trends and characteristics of the sampled populations in reference to their OHS use, deeper questions remain unanswered with respect to the meaning and implications of these findings, and especially with respect to recommendations for improvement. Our previous study provided a fair understanding of OHS use among older Israelis in terms of general demographics, socioeconomic status and gender. Similarly, based on that study we also gained a better understanding of what older adults do with OHS and when and where they access OHS in terms of their use of relevant technologies and their differential ICT-related skills. Barriers associated with effective OHS adoption among older adults have been examined using several leading theoretical technology adoption models. In our study, we merged the TAM (Technology Acceptance Model) [8, 9], the UTAUT (Unified Theory of Acceptance of Technology Model [22]), the HBM (Health Belief Model [23, 24]) and the HIAM (Healthcare Information Adoption Model [6, 7]) to gain insights regarding the central barriers associated with OHS adoption among older adults in Israel.

### Open questions

Despite the above findings, some open questions still remain. We recognized the need for a deeper understanding of issues related both to the barriers and to the catalysts of OHS adoption. For example, since 22% reported being “unaware” of the existence of OHS or of the benefits of its use, we felt the need for a more in-depth inquiry into the exact nature and causes of this unawareness. Another open question is related to the 32% of survey participants who reported feeling “uncomfortable” using OHS. What exactly do they mean by “feeling uncomfortable”? Are these feelings simply related to a general tendency toward technophobia known to be associated with older populations? Can they be attributed to some other hidden personal or psychological barriers? Or perhaps these uncomfortable feelings stem from more deeply rooted social norms related to expected patient-physician relations, as reported for example by Marton [25]. Finally, we sought to gain a better understanding of the role of family members in helping older adults overcome the barriers to OHS adoption, a subject that was constantly mentioned by the survey respondents. We felt a qualitative research study was needed to address these issue and open questions.

## Methods

We conducted a series of in-depth semi-structured open phone interviews with 31 individuals age 50 and up in Israel, as outlined below. The individuals selected for the interviews were picked from the sample reported in Shahrabani and Mizrachi [5] and thus are proportionally representative of the participants in the broad initial quantitative survey (21 users – 9 men and 12 women; 10 non-users – 4 men and 6 women). The proportions of Jews and non-Jews were similar. Moreover, non-users and users were also represented in proportion to the survey findings, as were age and gender and other relevant demographic variables. In terms of age, 12 respondents were between the ages of 50 and 54, six respondents were between the ages of 55 and 60, seven respondents were between 61 and 69, and six respondents were 70 years old and above. The interviews lasted 30 min on average and included mostly open-ended questions targeting two distinct populations: users who use at least one OHS, and non-users who do not use OHS at all. Questions for the first group were aimed at pinpointing the exact nature of their positive experiences using OHS as well as extracting information that may turn out to be useful in enhancing and replicating success factors in OHS use. Sample questions included: “What can your health care provider do to further encourage you to use OHS?”; “What can your medical support personnel do to further encourage you to use OHS?”; “What new and useful online services not offered today by your OHS provider can you think of and will you use them?” Questions for the second group were aimed at pinpointing the exact nature of older adults’ negative experiences with OHS as well as finding ways to mitigate the factors related to such failures. Sample interview questions for the older non-users included: “What exactly discourages you from using OHS?”; “When you state that you see no need to use OHS, what exactly do you mean?”; “What would convince you to use OHS?”; “What can your healthcare service provider do to encourage you to use OHS?”; “What new and useful online services not offered today by your OHS provider can you think of that will cause you to start using OHS?”

Based on generic observations by both Shanas [26] and Cantor [27] that “old people turn first to their families for help, then to neighbors, and finally, to the bureaucratic replacements for families because they expect families to help in case of need”, we thought a similar pattern might emerge regarding the role played by family members in OHS adoption among older adults. Accordingly, throughout the interviews, we continuously asked the interviewees to characterize the role of family members in furthering and supporting them in adopting OHS. Thus, for example, interviewees were asked to characterize the specific ways in which family members mediate their OHS use and to indicate whether family members helped improve their ICT skills, thus facilitating their OHS adoption.

Data were collected during the months of July and August 2014. Given the semi-structured nature of our qualitative interviews, the use of heavyweight qualitative analysis software packages with our data seemed excessive and redundant. Two of the researchers (Mizrachi and Nachamani – Team 1) identified central themes using more traditional word processing and charting methods. The other two co-authors (Shahrabani and Hornik – Team 2) independently validated and modified these themes. Team 1 began by reading the transcripts and, when relevant, the interviewer notes. After reading these transcripts several times and taking notes, Team 1 began color-coding the transcripts with a word processing program, using a different color for each emerging idea. The team consolidated groupings of repeated ideas into themes that were derived deductively, beginning with themes from the literature on technology adoption, as well as inductively by allowing themes to emerge from the data. Often the themes that emerged from these data differed slightly from those in the literature but were linked to or overlapped existing themes. To increase the study’s trustworthiness, Team 2 verified Team 1’s findings. After reviewing the participants’ transcripts and the interview protocol used during data collection, Team 2 confirmed the identified themes after making slight changes and modifications and provided additional insights into the texts and themes derived by Team 1. This type of analysis is equivalent to internal validity testing [28, 29] (Creswell 2007; Lincoln and Guba 1985).

## Results

Four major themes and primary observations emerged from our data:

1. While older adults are aware of OHS to some extent, they often do not fully understand the specific benefits of using these services;
2. Older adults need to acquire much more experience using OHS. OHS user interfaces still have a long way to go for older adults to feel comfortable using them. People age 50 and up seem to be less concerned about privacy and security issues than about seemingly more trivial issues such as recovering forgotten passwords;
3. Family members can play key roles in helping older adults adopt OHS by providing technical support as well as motivation and encouragement;
4. Based on their background and experience using analogue methods, older adults have worthwhile recommendations for innovation and policy improvement that would facilitate wider OHS adoption.

### Theme I: “This is not for me” – OHS awareness issues

One of the main reasons interviewees gave for not using OHS is that they are “unaware” of the existence and/or the benefits of OHS use. One common answer was: “I do not need to use such services.” Further investigation yielded a variety of reasons, including *“I have no need for OHS, I am healthy”* (most common statement) and *“OHS are not relevant for me.”*

Overall, these non-users seemed to lack any perspective on OHS as something that may be useful in terms of time saving, convenience and improved monitoring of medical information. They are “aware” that OHS exists, but they do not seem to see any specific potential benefits, even though older people clearly do have routine medical issues such as checkups, blood tests, and other geriatric-related issues. This failure to perceive any specific potential benefits of OHS has some important implications on how to best “market” OHS to older adults.

With respect to people age 50 and up who currently use OHS, we sought a more in-depth understanding of their behavioral patterns related to successful OHS adoption in order to replicate, transfer and enhance best practices. Hence, we asked participants in this group to focus more specifically on the major benefits they perceive in OHS use, thinking that their answers would help in future leveraging efforts with respect to non-users. In response, the absolute majority of OHS users in this group used the words “convenience,” “user-friendliness,” “helpfulness” and “efficiency” when describing why they use OHS. Interviewees were also asked to respond to open questions, such as: “How did you become aware that OHS is useful for you?”; “Do you think that other people in your social (and age) groups are not sufficiently aware of the advantages of such services?”

We also considered the possibility that traditional offline and online social networks used by people in this age group may also influence OHS awareness levels and serve to make recognition of OHS benefits go viral. Hence, we asked our non-user interviewees whether people in their social networks use OHS. If they answered in the affirmative, we asked them to describe what their peers told them about their experience using OHS. We were surprised to find that many respondents stated their peers use OHS. For example, one respondent stated: *“Many of my friends use OHS but I do not know if they are happy with it.”* Others gave even more specific answers: *“They use it only to find out about blood test results.”* These answers indicate that despite their fine-tuned awareness of OHS use among their peers, non-users consciously chose not to adopt OHS. Finally, and perhaps not surprisingly, older OHS users reported that most of their friends are very satisfied using OHS. Clearly, establishing a social circle of satisfied OHS users among older adults has the potential to instigate a positive loop toward OHS usage.

### Theme II: “I hate using those screens” – user interface and experience

In addition to their lack of awareness, many non-user interviewees in our quantitative survey reported feeling “uncomfortable” using OHS. We sought to discover what exactly they meant by feeling “uncomfortable.” Our open qualitative interviews revealed a wide range of reasons and barriers that inhibit OHS use among older users. As expected, some mentioned physical limitations: *“My vision is not as good as it used to be – I hate using those screens.”*

Two additional recurrent themes also emerged from the interviews:

#### Technical issues

The most common technical complaint was: *“I constantly forget my password and it is always – always - an issue getting a new one.”* Several interviewees complained that this issue is particularly troubling in the OHS context:

*…"every time that happens, I have to go to the clinic in person or else use my phone for text messages in combination with the browser… on other sites this is so much easier. It's about time they change this!"*

It seems that greater concerns about privacy issues in the OHS context make supposedly simple identification and authentication processes more complicated, especially for older users. Yet the majority of the interviewees did not see privacy issues as a major concern. Here are two examples: *“I have no secrets”; “Who would want to know an old man’s medical issues?”* These findings point to the need for easier online authentication and identification procedures for the older adult population segment.

#### Unwillingness to change old habits

“Laziness” was repeatedly cited in the interviews as a common reason for not using OHS. We found this to be somewhat surprising as the whole idea behind OHS is to make things easier for users. Further inquiry into the nature of this “laziness” revealed that the real issue is that “old habits die hard,” perhaps especially among older people. Hence, typical answers to the follow-up questions included:

*"I am used to making appointments on the phone with a real person who actually talks to me."*

*"Virtual appointment setting on the browser does not feel real. I always worry that this appointment was not actually made."*

One interviewee captured the essence of this theme by stating:

*"It is difficult for me to change my old habits when it comes to appointment scheduling. This is both because of my old routine in doing things and perhaps even my own [personality] character. At my age, it is hard to adapt to new ways of doing things, especially if they worked for you throughout your life."*

### Theme III: “Let me call my grandson” – the key role of family members

For all the above issues, interviewees were asked to comment on the potential role of family members in improving their OHS adoption. In order to gain specific, well-defined and applicable answers with the potential to be used for recommendation purposes, we had to break down the broad notion of OHS into specific domains that our interviewees could comment on. The professional literature includes several typologies and classifications of OHS or Consumer e-Health Applications [30]. For the purposes of this study, we distinguished two generic types of online health services: 1) Consumer Health Informatics or Electronic Health Records, referring to the use of information and communication systems to collect, analyze and distribute medical information; and 2) Telemedicine or TeleHealthcare, referring to information and communication systems that combine hardware components designated for surveillance, data analysis and remote treatment of patients. Our open interview questions focused on the first type of online health services, which are more common and more advanced. We further focused our questions on the following three types of OHS already offered by all of Israel’s main HMOs as well as most public hospitals:

1. Formal administrative and content-related medical information (with a one-sided or two-sided interactive, formal and institutional emphasis) from medical institutions, such as appointment scheduling, lab test results, interactive guides and blogs, and continuous mobile-based pregnancy monitoring.
2. Informal content-related medical information (with a two-sided, interactive, informal and non-institutional emphasis). This information comes from content-related websites, such as forums and medical information communities, independent blogs and blogs sponsored by pharmaceutical companies and private institutions.
3. Online medicine at home, such as monitoring systems that use designated hardware (for blood pressure, pulse and sugar level monitoring) to report back to institutional treatment information systems via internet or mobile networks, for example by remotely activating and controlling designated appliances for chronic illnesses and geriatrics.

Using the above distinctions enabled us to better pinpoint and delineate the role played by family members with respect to OHS adoption among their older relatives. In the case of non-users, interviewees assisted by family members reported that their relatives use “the computer” on a bi-weekly basis to assist them. This assistance primarily involves matters of formal administration and content-related medical information, such as appointment scheduling and obtaining lab test results. Most of the interviewees indicated that this is “very helpful” though they do not plan to use OHS themselves for the reasons outlined above. Among those who do not get help in using OHS from family members, when asked whether they would be willing to accept such assistance, typical answers included: *“I am not sure”* or “*I do not need this kind of help.”*

Such answers may perhaps hint at some deeper interpersonal issues within the family unrelated to OHS utilization, especially given the somewhat ironic emphasis on “this kind of help” as opposed to other forms of family assistance. Those who hoped family members would use OHS indirectly to assist them with health issues seemed to exhibit a fair understanding of the benefits of OHS. Yet they remained passive both with respect to adopting OHS directly and regarding seeking family support on these issues.

A few of the interviewees also commented on issues related to informal content-related medical information and the role family members may play in this realm. One interviewee, for example, mentioned she hoped someone in her family would assist her in finding “a specialist doctor” for consulting purposes on some dedicated online medical forum since she is “very confused and frustrated” by the conflicting answers “Dr. Google” provides. She noted that this confusion is what “I hear from my [elderly] friends that use the internet.”

Specific questions also addressed the role of family members in promoting OHS adoption. Thus, for example, interviewees were asked to characterize the specific ways in which family members mediate OHS for them. Most of the older OHS users reported that their family members also use OHS and tell them they are “very happy with these services” and they (the older users) “must start using them.”

In fact, older OHS users reported that this kind of “push” from family members to become acquainted with and start using OHS was a “turning point” in their OHS adoption. Here, too, we see that positive encouragement from a family member to use OHS may serve as a catalyst for successful OHS adoption among older adults. It is interesting to note that people age 50 and up who have already adopted OHS and use it on a daily basis rarely seek family support on these matters. Based on our interviews, it seems that once a person has crossed the “OHS usage Rubicon,” the role of family members as OHS ambassadors and assistance providers diminishes dramatically.

### Theme IV: policy and innovation – “Advice from the Analogue Generation”

In our efforts to gain a better understanding of the “non-users”, we sought to derive some deeper insights from a series of questions related to future strategy formulation and recommendations. For example, we sought to understand what would convince these older “non-users” to adopt OHS, and more specifically, what improvements OHS providers should make to encourage them to do so. We already mentioned the important role of family members in encouraging OHS adoption. To extrapolate, we further sought to discover what medical teams in hospitals and healthcare clinics could do to persuade these “non-users.” To this end, we asked this question openly and bluntly. Most of the interviewees emphasized the steps they themselves must undertake. One interviewee stated:

*"Here is a piece of advice from my analogue generation. I do not need persuasion; I already know that OHS are important. I just need to motivate myself and take the time to learn how to use this technology."*

One interviewee even made the following interesting and humorous observation that we believe captures an important insight:

*"They should pay me every time I use the computer to contact them. When they do this I'll reconsider."*

We think this is not a bad idea. Incentive programs to boost OHS use among older populations should consider adding a rebate component to facilitate usage. Such a step would optimize use of the physical, time and space resources at health service provider clinics. Indeed, the benefits accruing to the health service provider from the improved data collected for predictive and analytic purposes might very well justify any minor financial incentive used to encourage older adults to use OHS.

In contrast to non-users of OHS, the users among our research population were much more eager and enthusiastic about finding new ways to “convert” non-users and “recruit them to our camp.” In the interviews they were asked the following question: “In your experience, what persuasion efforts can medical teams in hospitals and healthcare clinics make to accelerate OHS adoption?” Many had lots of ideas, and some were practically evangelists in their reactions. The most common answer was: “Talk to their family members and ask them for their help on this matter”.

The participants had other interesting suggestions that we believe qualify them as the best ambassadors for promoting OHS among non-users. In the following representative set of responses, user interface improvement seems to be a highly important and recurring theme for accelerating OHS adoption: *“Make the fonts bigger”; “Cancel the mandatory bi-monthly password renewal procedure”; “Provide support in additional languages so that more users will use the site.”*

Another recurring theme was the need to increase awareness of available services and their benefits: *“Inform people about new services via email and text messages”; “You must advertise more! People do not know about it. What a waste.”*

The older users in our sample also indicated that another major incentive for future rapid and large-scale adoption is to have healthcare providers increase the range and frequency of advanced online services. In other words, the ROI for OHS usage among older users must be improved. Common examples included adding appointment scheduling for para-medical services (e.g., occupational therapy, physiotherapy, holistic health services such as acupuncture) as well as providing automated drug prescription services for chronic patients: *“I will be taking these pills till the day I die so why must I ask for them again and again?”* Other suggestions included offering instant emergency calls via the internet, replacing old fax authorizations with emails and more.

On issues related to future telecare services being developed by many OHS providers, we sought to better understand the dynamics and active forces behind possible future adoption of such services among older adults. We asked the interviewees open questions such as the following: “Under what circumstances would you agree to consult specialist doctors in a web-conference setting?”; “Which (currently unavailable) telecare services would you feel comfortable using?” We hypothesized that this last question together with a list of specific possible cases for reference could be helpful in assisting OHS providers to better understand the likelihood of possible adoption of innovative telecare services in the future. Once again, the non-users were much less helpful in this regard, while the OHS users were eager to answer these open questions, gave innovative responses and expressed their willingness to experiment with the proposed upcoming services, among them remote online heartrate and blood pressure monitoring, online video conferencing with specialist doctors, remote telepathology and teleophthalmology diagnostic services and more. When asked about possible technical barriers to using these new services, the common answer was: *“In such a case, I will ask someone from the family to assist me.”* This answer is in line with our other findings.

## Discussion

The semi-structured and open-ended data collection format employed in the qualitative research framework reported here enabled us to discover important nuances with respect to OHS adoption barriers among people age 50 and up. Such nuances are usually not revealed when the research approach includes only quantitative, survey-based closed-question questionnaires. We began by examining awareness—the primary enabler of any OHS adoption move. The qualitative findings show that while most of the interviewees were generally aware of the potential value of OHS, this awareness did not necessarily translate into action (OHS adoption) because they were unaware of the concrete, specific and tangible benefits of these technologies. Hence, and in accordance with the literature, the key element of perceived benefit—so critical to any technology adoption—is not optimally satisfied in the case of older populations. Perceived benefit is defined as consumers’ belief about the extent to which they will become better off using a certain online service [29]. Evidence from prior studies has shown that perceived benefit exerts a positive and significant effect on customers’ behavioral intentions [31, 32]. OHS are among the many internet-based services that offer potential benefits to consumers such as cost and time saving [33] that have been identified as relative benefits compared to those of traditional offline services [34]. Accordingly, if individuals perceive the benefits are greater, they are more likely to adopt OHS. Hence, our findings may help online healthcare policymakers identify the critical divide between the perceived potential general benefits of OHS and the immediate and concrete sense of perceived benefit grounded in specific cases. This concrete sense of perceived benefit is critical in any OHS adoption move, especially among older populations more oriented to analogue actions.

Our findings also seem to support the notion that what younger people perceive as older people’s “tactical” and “minor technical” concerns regarding OHS adoption are in fact often seen by older people as real and almost tangible barriers. This finding is also in line with observations reported by Folkman and Lazarus [35], who suggested that stress arises when individuals experience certain situations or events as threatening or demanding and believe they lack the appropriate skills and coping resources for handling such situations. Stawski et al. [36] also emphasized the importance of understanding the role of age in exposure and reactions to daily hassles. Such hassles have been found to cause psychological distress, impede wellbeing and have negative health outcomes among older people [37]. Indeed, research indicates that age plays an important role both in exposure and in reactivity to daily stressors. Older adults report a stronger preference for avoiding daily stressors than young people, perhaps because they react more emotionally to the stressors they do experience [36]. Thus, the stress experienced by older adults when using OHS appears to play an important role in their inclination to use OHS or to avoid these services altogether. Finally, the psychological outcome of stressful encounters is determined by the interaction between the nature of the stressor, the individual’s resources such as social support or self-efficacy and the individual’s own appraisals of the stressor [38].

As noted by Conrad et al. [39], caregivers, patients and their families increasingly use online resources for health information, in addition to seeking out traditional sources. In fact, as shown by a recent survey by the Pew Research Center [40], searching for health and healthcare information is the third most common online activity, with nearly three quarters (72%) of adult internet users seeking health support and information on the internet. When it comes to social media, online health information-seeking continues to gain momentum as well, with new interactive tools increasingly being adopted [39]. Thus, with 67% of all internet users using social media [41], 26% use these new media tools for exploring health-related issues or reading about someone else’s health experience online [42]. Our findings that privacy issues seem to be of lesser importance is well in line with recent findings by Rock Health [43] showing that while consumers are concerned about the privacy and security of their health data, the vast majority (77%) are interested in sharing their health information, especially to get better care from their doctor.

Our findings about the importance of guidance and support from family members in accelerating OHS use also echo observations made by Marton [25], who developed and tested a theoretical model of online HISB (Health Information Seeking Behavior) based on an exploratory multi-method study of 264 older women. In her model, Marton found two situational factors—health condition and family support—to be positively related to HISB frequency on the web. The participants’ mixed reactions regarding how to share and challenge their doctors with healthcare information they read about on social networks and dedicated online forums echo Marton’s [25] findings. In both studies, older users tended to place their faith in their physician’s expertise and felt that information-seeking would be perceived as violating their role as patient, reflecting the Parsonian sick role theory [44].

## Conclusions

Although research suggests that older people tend to use technology, including OHS, less frequently than younger adults [38, 45–47], the obstacles to OHS use among older adults can be overcome when family members take an active role in bridging efforts. The findings reported here are quite compatible with contemporary literature on the central role of family members in encouraging and mediating OHS use among their older relatives [25]. Indeed, older people are the primary beneficiaries of OHS, and their widespread OHS usage has the potential to make health care services more accessible to them and also to reduce the burden on the health system.

While this study has not focused on any specific/tactical usability issues, several recurring insights related to OHS usability emerged from our interviews with people age 50 and up. While the older interviewees did not use UI-UX (UI-User Interface, UX-User Experience) nomenclature to express their frustrations in their digital encounters, they repeatedly raised the following two issues: 1) issues of text and icon readability (e.g., fonts and icons: the bigger, the better; color and contrast issues); 2) issues regarding text and icon comprehension and the need for speech compliance solutions and coherent semantics adjusted to older people (e.g., Text to Speech (TTS) solutions and the need to acknowledge that phonetics, slang, and wordplay can present challenges to certain age groups). The technical solutions to these issues should ultimately be provided by UI-UX design experts. We feel that any solution must be tested extensively, for this the only real way to gain insights into the cognitive processes and physical limitations of older people as well as to determine which parts of the UI system need re-engineering and improvement.

Based on the findings of this study, we also recommend that HMOs increase media channel advertising to emphasize the benefits of OHS use. These advertisements should target older adults and their families. In addition, HMOs should improve their OHS websites to make them more accessible and user-friendly for older people. These websites should offer the possibility of contacting helpdesk advisors trained to help older users. Likewise, for adults age 65 and older who use the internet, HMOs can offer short comprehensive video clips that clearly explain how to use various OHS. Moreover, HMOs should offer community OHS guidance programs at their facilities for older adults who use the internet. These measures may help narrow the gap between required skills and user competencies, thus enabling older adults to benefit from OHS tools.

With respect to the group of older adults age 70 and above who do not use OHS at all, we recommend that young people help and train these people to use OHS as part of their national service in Israel. Another recommendation relevant to this age group of non-users is to train HMO nursing teams (e.g., in remote medicine technology units) to contact older adults and their caregivers and promote OHS use among them. Indeed, with the rapid development and spread of remote medicine technology during and after the COVID-19 pandemic, promoting OHS use among non-users has become especially important.

## Data Availability

The dataset supporting the conclusions of this article is available from the authors upon request.

## Abbreviations

HBM: Health Belief Model
HISB: Health Information Seeking Behavior
HMO: Health Maintenance Organizations
HIAM: Healthcare Information Adoption Model
ICT: Information and Communications Technology
OHS: Online Health Services
ROI: Rate On Investment
TAM: Technology Acceptance Model
UTAUT: Unified Theory of Acceptance of Technology
UX: User Experience
UI: User Interface

## Footnotes

1 In the U.S., for example, the share of non-internet users ages 65 and older is 27% compared with fewer than 10% of adults under the age of 65. https://www.pewresearch.org/fact-tank/2019/04/22/some-americans-dont-use-the-internet-who-are-they/
